# Temporal changes in HCV genotype distribution in three different high risk populations in San Francisco, California

**DOI:** 10.1186/1471-2334-11-208

**Published:** 2011-08-02

**Authors:** Paulo Telles Dias, Judith A Hahn, Eric Delwart, Brian Edlin, Jeff Martin, Paula Lum, Jennifer Evans, Alex Kral, Steve Deeks, Michael P Busch, Kimberly Page

**Affiliations:** 1 Núcleo de Estudos e Pesquisas em Atenção ao Uso de Drogas (NEPAD)- Universidade do Estado do Rio de Janeiro (State University of Rio de Janeiro), R Fonseca Teles 121, 4°. Andar; 20940-200 Rio de Janeiro, RJ, Brasil; 2 Department of Epidemiology and Biostatistics, Division of Preventive Medicine and Public Health, Box 1224, 50 Beale Street 1200 University of California San Francisco, San Francisco, CA USA; 3 Department of Medicine, Division of Infectious Disease, San Francisco General Hospital, Box 0811, SFGH Bldg 100 335; University of California San Francisco, San Francisco, CA. 94143 - 0811, USA; 4 Blood Systems Research Institute, 270 Masonic Avenue, San Francisco, CA 94118, USA; 5 Department of Medicine, SUNY Downstate College of Medicine, 450 Clarkson Avenue, Box 1240, Brooklyn, NY 11203. USA; 6 Department of Epidemiology and Biostatistics, Division of Clinical Epidemiology, Box 0560, 185 Berry Street 5700, University of California San Francisco, San Francisco, CA. 94143 - 0560, USA; 7 Department of Medicine, Positive Health Program, San Francisco General Hospital, Box 0874, SFGH Bldg 100 335; University of California San Francisco, San Francisco, CA. 94143 - 0811, USA; 8 Research Triangle Institute (RTI), San Francisco, CA USA; 9 Department of Laboratory Medicine, Box 0134, University of California San Francisco San Francisco, CA. 94143 - 0134 USA

**Keywords:** hepatitis C virus, HCV, GT, injection drug use, HIV, birth cohort, African-American

## Abstract

**Background:**

Hepatitis C virus (HCV) genotype (GT) has become an important measure in the diagnosis and monitoring of HCV infection treatment. In the United States (U.S.) HCV GT 1 is reported as the most common infecting GT among chronically infected patients. In Europe, however, recent studies have suggested that the epidemiology of HCV GTs is changing.

**Methods:**

We assessed HCV GT distribution in 460 patients from three HCV-infected high risk populations in San Francisco, and examined patterns by birth cohort to assess temporal trends. Multiple logistic regression was used to assess factors independently associated with GT 1 infection compared to other GTs (2, 3, and 4).

**Results:**

Overall, GT 1 was predominant (72.4%), however younger injection drug users (IDU) had a lower proportion of GT 1 infections (54.7%) compared to older IDU and HIV-infected patients (80.5% and 76.6%, respectively). Analysis by birth cohort showed increasing proportions of non-GT 1 infections associated with year of birth: birth before 1970 was independently associated with higher adjusted odds of GT 1: AOR 2.03 (95% CI: 1.23, 3.34). African-Americans as compared to whites also had higher adjusted odds of GT 1 infection (AOR: 3.37; 95% CI: 1.89, 5.99).

**Conclusions:**

Although, HCV GT 1 remains the most prevalent GT, especially among older groups, changes in GT distribution could have significant implications for how HCV might be controlled on a population level and treated on an individual level.

## Background

Hepatitis C virus (HCV), first identified in 1990, is a single strand RNA virus in the family *Flaviviridae*. It is prone to high rates of genetic mutation, resulting in evolution to divergent forms and six major genotypes [1]. Each genotype (GT) is further divided into subtypes, based on genetic sequence variability. There are notable clinical differences in responsiveness to interferon-based therapy for treatment of chronic infection by GT, with GTs 1 and 4 being less responsive and requiring longer exposure time to treatment than types 2 and 3 [2-5]. GT has been associated with different patterns in HCV viremia during interferon treatment [6], and antiviral resistance [7,8]. Although some clinical conditions have been noted to differ by HCV GT, including insulin resistance [9], HIV and HIV disease progression [10], little is known regarding how different HCV GTs differ in virulence or pathogenicity [11]. African Americans infected with GT 1 are significantly less likely to respond to HCV treatment than Asian and Caucasian patients with GT 1 [12,13]. Patients infected with GT 1 who carry a genetic variant allele near IL28B gene are more likely to respond to combination therapy (pegylated interferon-alpha-2a or -2b and ribavirin) [14] and clear spontaneously [15]. As this polymorphism varies by race [14], it partially explains differences in these outcomes. Finally, GT heterogeneity may hinder the development of a vaccine [16].

In the U.S. HCV GT 1 has been the most common infecting subtype; over 70% of patients with chronic infection are GT 1 [17-19]. In Europe, however, recent studies have reported more variation in the extent and diversity of HCV GTs, and have shown associations between HCV GT and risk exposure, age, and clinical group [20-24]. In the U.S., race/ethnicity have been found to be independently associated with GT in the NHANES general population sample [19] and in other samples [18], but none have examined temporal changes in circulating infecting GTs.

The objective of this study was to examine the distribution of prevalent HCV GTs in various high risk populations in San Francisco by birth cohort and other demographic factors.

## Methods

We examined data from three prospective cohort studies in San Francisco, California, U.S. In total, 460 chronically HCV infected persons were included from the following studies, all with IDU exposure: 1) The "Urban Health Study" (UHS), a prospective serial cross-sectional study of IDU recruited from San Francisco neighborhoods with high prevalence of IDU[25]; 2) The UFO Study, a prospective cohort study on the incidence of HCV infection in young (<30 years) street-recruited IDU[26]. 3) The "Study of the Consequences of the Protease Inhibitor Era" (SCOPE) Study, a prospective, observational study of HIV positive patients[27]. Subjects included in this study were recruited over varying intervals during a twenty-year time period between 1987 and 2007 (Table 1). HCV GT was assessed in the participants with incident HCV infection in the UFO Study and the UHS using stored sera specimens from blood samples taken at the visit of first HCV detection (anti-HCV or HCV RNA). In the SCOPE study HCV GT was determined from stored samples of prevalent infection obtained at first or second study visit (if not available from the first study visit).

**Table 1:** Selected characteristics of participants in three studies of HCV infection in San Francisco (N = 460)

Variables	UHS	UFO	SCOPE	Total all studies
Total N	202	128	130	460

Gender N(%)¶				
Male	145 (72.5%)	88 (68.8%)	85 (65.4%)	318 (69.4%)
Female	55 (27.5%)	40 (31.2%)	45 (34.6%)	140 (30.6%)

Age (in years)#				
Median	47.0	22.3	45.0	43.0
IQR	42.3-50.0	19.9-25.0	40.8-49.0	28.0 - 48.0

Years of recruitment	1987-1998	2000-2007	2000-2007	1987-2007

HCV Genotypes* ¶			95 (77.9%)	326 (72.4%)
Type 1	161 (80.5%)	70 (54.7%)	10 (8.2%)	50 (11.1%)
Type 2	22 (11.0%)	18 (14.1%)	15 (12.3%)	69 (15.3%)
Type 3	15 (7.5%)	39 (30.5%)	2 (1.6%)	5 (11.1%)
Type 4	2 (1.0%)	1 (0.8%)	122 (100%)	#***
	#*		#**	

HCV Genotypes*¶				
1	15 (7.4%)	32 (25.0%)	62 (47.7%)	110 (23.9%)
1a	76 (37.6%)	26 (20.3%)	3 (2.3%)	105 (22.8%)
1a/1b	5 (2.5%)	1 (0.8%)	4 (3.1%)	10 (2.2%)
1b	65 (32.2%)	11 (8.6%)	26 (20.0%)	101 (22.0%)
1/2	1 (0.5%)	0 (0.0%)	0 (0.0%)	1 (0.2%)
2	1 (5.0%)	5 (3.9%)	0 (0%)	5 (1.1%)
2a	0 (0.0%)	1 (0.8%)	0 (0.0%)	1 (0.2%)
2a/2c	4 (2.0%)	6 (4.7%)	2 (1.5%)	12 (2.6%)
2b	17 (8.4%)	6 (4.7%)	3 (2.3%)	26 (5.7%)
2b/3a	1 (0.5%)	0 (0.0%)	0 (0.0%)	1 (0.2%)
3a	15 (7.4%)	39 (30.5%)	15 (11.5%)	69 (15.0%)
4	1 (0.5%)	0 (0.0%)	1 (0.8%)	4(0.4%)
4c/4d	1 (0.5%)	1 (0.8%)	1 (0.8%)	3(0.7%)
Undetermined	0 (0.0%)	0 (0.0%)	8 (6.2%)	8 (1.7%)**

Ethnicity¶				
Caucasian	55 (27.5%)	97 (75.8%)	54 (41.5%)	205 (44.8%)
African Amer.	124 (62.0%)	2 (1.6%)	54 (41.5%)	180 (39.3%)
Latino	10 (5.0%)	7 (5.5%)	9 (6.9%)	26 (5.7%)
Asian	1 (0.5%)	1 (0.8%)	3 (2.3%)	5 (1.1%)
Other	10 (5.0%)	21 (16.4%)	10 (7.7%)	42 (9.2%)**

Education (1)¶				
< High School	56 (28.0%)	67 (52.8%)	34 (26.2%)	157 (34.4%)
>= High school	144 (72.0%)	60 (47.2%)	96 (73.8%)	300 (65.6%)**

* Co-infected cases not included.

** Statistically significant difference (p < 0.05)

# Age at the time of the interview

¶ Some cells do not conform to the total sample size totals due to missing data

#* 2 people co-infected with different genotypes were not included

#** 8 people with undetermined genotypes were not included

#*** 10 people not included due to undetermined genotypes or to co-infection with different genotypes

In all studies anti-HCV and HCV viremia were assessing using Ortho EIA 3.0, with confirmation by RIBA 3.0, Chiron, Corp, and a discriminatory HCV transcription-mediated amplification (dHCV TMA) assay component of the Procleix HIV-1/HCV assay (Novartis Diagnostics, Emeryville, CA, USA), respectively. HCV GT was determined by the LiPA Line Probe Assay (Bayer Diagnostics, Tarrytown, NY, USA). Indeterminate GTs and co-infected cases were not included in the final analysis of the study.

We assessed differences between the study groups and between GT 1 versus other GTs with respect to sociodemographic and clinical variables using standard methods. Differences in proportions were compared using Pearson Chi-Square tests. Differences in variables with continuous data were assessed by comparing medians and distributions using the Kruskal-Wallis test. We examined HCV GT distribution by birth cohort to assess trends by 10-year birth intervals using the Mantel-Haenszel Chi-Square test for trend. Multiple logistic regression was used to assess factors independently associated with GT 1 infection compared to other GTs (2, 3, and 4); adjusted odds ratios (AOR) and 95% Confidence Intervals (CI) were calculated. Models were constructed in an iterative backwards stepwise manner. All variables with a p-value <= 0.05 at the bivariate level were entered into the model and removed one by one. Variables with a p-value of <= 0.10 were retained in the final model. All analyses were conducted using SPSS for Windows statistical software package (version 13).

All of the research from participating studies in this project was conducted under protocols reviewed and approved by the University of California San Francisco Human Subjects Research Committee, and conformed to the Helsinki Declaration and local regulations. All study subjects gave informed consent for participation.

## Results

**Figure 1:**
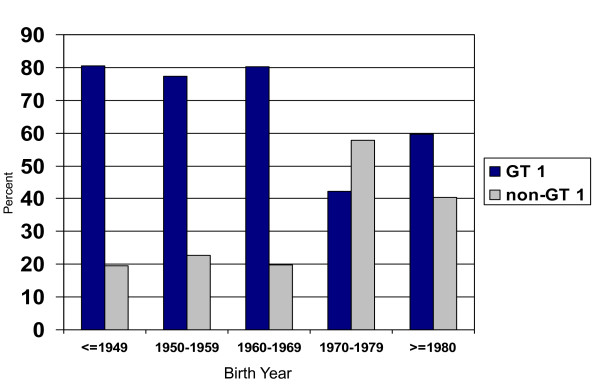
**Distribution of HCV genotypes 1 and non-1 among three high risk populations in San Francisco by birth cohort (decade)**.

Of the 460 persons included, 202 (43.9%) were from the UHS; 128 (27.8%) from UFO; and 130 (28.3%) from the SCOPE studies. Table 1 shows participant characteristics by study. There were significant differences in age, race/ethnicity and educational background between the three groups, and males comprised the majority (69.4%) in all. GT 1 was predominant (72.4%) overall, however between groups the distribution of GTs was significantly different. Young IDU (the UFO Study) had the lowest proportion of GT 1 infections (54.7%) compared to older IDU (UHS) and HIV-infected patients (SCOPE) who had 80.5% and 76.6%, respectively. Analysis by birth cohort also showed significant differences (see Figure 1). The more recent birth cohorts had increasing proportions of non-GT 1 infections compared to less recent birth cohorts, and young IDU specifically had more GT 3 infections compared to others (Table 1). Table 2 shows the bivariate and multivariate results of variables associated with GT 1 infection compared to GTs 2, 3, and 4 (non-GT 1) infection. Factors independently associated with prevalent infection with HCV GT 1 included birth cohort and race/ethnicity. Patients born before 1970 had higher adjusted odds of GT 1 infection compared to those born more recently: AOR 2.03 (95% CI: 1.23, 3.34); age and study group were not included in the final model due to colinearity with birth cohort. African-Americans compared to Caucasian patients were more likely to have GT 1: AOR 3.37 (95% CI: 1.89, 5.99).

**Table 2:** Bivariate and multivariate associations between selected demographic characteristics and infection with HCV genotype 1 vs. non-1 (genotypes 2, 3, 4)

	% with genotype 1 HCV infection	Odds Ratio (95% CI)	p-value	Adjusted# Odds Ratio (95% CI)	p-value
Gender					
Male	73.4	1.22 (0.78, 1.90)	0.38		
Female	69.3				
					
Ethnic Background					
Caucasian	59.2	1		1	
African American	87.1	4.65 (2.75, 7.86)	< 0.001	3.37 (1.89, 5.99)	<0.001
Latino	72.0	1.77 (0.71, 4.45)	0.22	1.57 (0.61, 3.98)	0.35
Other	62.8	1.16 (0.59, 2.30)	0.66	1.85 (0.59, 2.37)	0.63
					
Birth Cohort					
Born before 1970	79.0	2.97 (1.20, 4.59)	< 0.001	2.03 (1.23, 3.34)	0.006
Born 1971 or later	56.0			1	
Study group					
UFO	54.7	1		**	
UHS	80.5	3.33 (2.04, 5.45)	< 0.001		
SCOPE	76.7	2.92 (1.68, 5.06)	< 0.001		

** study group was not included in the model as [UFO vs. (UHS and SCOPE)].

# variables included in the final model were: Ethnic Background and Birth Cohort

## Discussion

These results suggest that in San Francisco, within the ongoing HCV epidemic, the epidemiology of infecting HCV viruses has changed over time. Among younger IDU, who are also more likely to have more recent infection, non-GT 1 is increasingly prevalent. Historically, few studies have been published describing GTs in San Francisco patients. In 1999, Gish et al, [28] described GTs (using restriction fragment length polymorphism analysis) in 186 patients with chronic HCV (the majority presumptively IDU); 76% were type 1 (1a, 41%; 1b, 35%), 13% were type 2 (2a, 3%; 2b, 10%) and 11% were type 3a. In that sample, HCV GT was not associated with age, gender, ethnic origin, or presumptive mode of transmission. In two small groups (n = 8 each) of chronically HCV infected, active and former IDU recruited from two urban San Francisco Hospitals, 75% of active IDU (median age 47 years) and all of the former IDU (median age 49) were type 1. As patients in these studies were more heterogeneous with respect to routes of exposure, and were likely to have been infected some years before clinical assessment, these data are consistent with the suggestion that type 1 GTs were more prevalent in San Francisco IDU in the 1990's.

In Europe, Simmonds et al [29] showed an association between HCV GT and age, and attributed it primarily to risk exposure differences. In Germany, Schroter et al [24] also showed increases in GTs 3a in younger groups, including IDU and non-IDU, leading these authors to hypothesize that the introduction of new subtypes in a high risk group may rapidly impact lower risk groups. Others have shown differences in HCV GT distribution by route of exposure, with GT 3a associated with injection exposures, and GT 1b associated with acquisition through blood transfusion [21,22,30,31]. In a multicentre study of HCV infection in 183 HIV-infected IDU sampled between 1984 and 2001, Van Asten et al [32], using phylogenetic analyses, found that subtypes 3a and 1a were most prevalent (38.3%, and 36%, respectively) and extensively dispersed throughout the countries.

The relative differences in HCV GT seen in these San Francisco patients may be due to various factors, including temporal changes in the dominant circulating virus, or potentially separate epidemics marked by differential social mixing. All three populations were sampled in San Francisco and were at high risk of HCV due to parenteral exposure, but younger patients had significantly lower odds of being infected with GT 1. African-Americans represented 62% of older IDU, only 1.9% of younger IDU, and 41.6% of HIV co-infected patients, but controlling for birth year and other factors, still had significantly higher odds of subtype 1 HCV infection compared to Caucasian patients. This differential GT distribution by racial group may be due to various factors including temporal, social (mixing), differential access to prevention, and/or possibly genetic explanations. The prevalence of IDU has decreased among younger age groups and among African-American populations in the U.S.[33], and drug injector networks and associated risks may influence the spread of HCV GTs [34,35] as seen with other diseases [36]. Risk groups, in particular IDU compared to non-IDU populations, may have differential access to prevention methods by age, race or other factors that might result in differences in HCV transmission patterns or detection [37-39]. The genetic polymorphism recently associated with treatment and spontaneous clearance of HCV [14,40] occurs with less frequency in African populations, and may account for some of the relative differences seen in HCV GTs prevalence in African Americans and possibly other racial/ethnic groups [19].

To our knowledge no studies have examined GT distribution patterns over several age groups (or by birth cohort) and risk group. In Spain, Serra et al (40), found that GTs varied by risk group: IDU were less likely to be infected with GT 1b and more likely to be infected with GTs 1a and 3, compared to transfusion recipients and blood donors, and GT prevalence varied over time in all groups [41]. Our results are also consistent with others who have shown that African-Americans are more likely to be infected with HCV GT 1 [18,19]. Significantly, a very recent report analyzing over 22,000 samples from HCV infected patients at a national reference laboratory in the U.S., found that patients infected with GT 3 were significantly younger (*P*<0.0001) than those with non-GT 3 (1, 2, or 4). the frequency of HCV GT 3 ranged from just 4.3% in subjects over age 70 to 20.6% in those age 21 to 30 [42]. Our results are consistent with these new findings, and among the first to suggest that the relative prevalence of GT 3 may be increasing as younger groups are more likely to have been recently infected.

This study has some limitations that should be acknowledged. Genotyping was performed by reverse hybridization, and viruses were not sequenced; misclassification could have occurred [43]. Patients may also be infected with multiple GTs, especially IDU who have frequent opportunities for exposure [44], and the assay would have only identified the dominant virus [45]. The groups here are selected from studies of high risk populations in San Francisco, so results may not be generalizable to other risk groups. Finally, two groups (UHS and SCOPE) predominantly represent chronically infected patients, and samples were genotyped only if HCV RNA was present. If there is differential spontaneous clearance in association with HCV GT [26], these results would not reflect the circulating infecting GTs in acutely infected patients, but only those in chronically infected groups.

## Conclusions

IDU are the population most at risk for HCV in the U.S. and younger IDU are most likely to present with more recently acquired infection. Although GT 1 remains the most prevalent GT, especially among older groups, changes in GT distribution coupled with changes in the demography of injection drug use [33] could have important implications for the HCV epidemic. From a public health perspective, these findings might have significant implications for how blood borne virus infections could be controlled on a population level and how they are treated on an individual level.

## Competing interests

The authors declare that they have no competing interests.

## Authors' contributions

All authors made substantive intellectual contributions to the study, including to conception and design, or acquisition of data, or analysis and interpretation of data, and drafting or revising the manuscript. All authors read and approved the final manuscript. PTD participated in the study conception, data analysis and drafting of the manuscript. JAH participated in study design, acquisition and analysis of data, and manuscript revisions. ED, BE, JM, ST, PL, JE, AK, SD and MB participated in study design, data acquisition, and drafting the manuscript. KP conceived of the study, participated in data collection and analyses, and the drafting and finalizing of the manuscript.

## Pre-publication history

The pre-publication history for this paper can be accessed here:

http://www.biomedcentral.com/1471-2334/11/208/prepub
